# Changes in Lipids in Granulomatosis with Polyangiitis Relates to Glucocorticoids and History of Hypertension

**DOI:** 10.3390/metabo13101053

**Published:** 2023-10-06

**Authors:** Marialuisa Sveva Marozzi, Antonio Vacca, Vanessa Desantis, Teresa Panebianco, Cristiana Catena, Gabriele Brosolo, Silvia Noviello, Anna Cirulli, Antonio Giovanni Solimando, Leonardo Alberto Sechi, Sebastiano Cicco, Roberto Ria

**Affiliations:** 1 Unit of Internal Medicine “Guido Baccelli”, Department of Precision and Regenerative Medicine and Ionian Area—(DiMePRe-J), University of Bari Aldo Moro, 70124 Bari, BA, Italy; 2 Unit of Hypertension “A.M. Pirrelli”, Department of Precision and Regenerative Medicine and Ionian Area—(DiMePRe-J), University of Bari Aldo Moro, 70124 Bari, BA, Italy; 3 Clinica Medica, Department of Medicine, University of Udine, 33100 Udine, UD, Italy; 4 Pharmacology Section, Department of Precision and Regenerative Medicine and Ionian Area—(DiMePRe-J), University of Bari Aldo Moro, 70124 Bari, BA, Italy

**Keywords:** cardiovascular risk, cholesterol, granulomatosis with polyangiitis, inflammation

## Abstract

Granulomatosis with polyangiitis (GPA) is an ANCA-associated small-vessel vasculitis. Vessel wall inflammation induces multiple vascular damages, leading to accelerated atherosclerosis. Metabolic profile and cardiovascular risk are somewhat understood in GPA patients. Cardiovascular atherosclerotic disease (ASCVD) may represent a risk for outcomes. Our purpose is to evaluate ASCVD risk in GPA patients. Thirty-six patients received GPA diagnosis (T0) and were evaluated after 1 (T1) and 2 (T2) years follow-up. All patients were treated with high-dose glucocorticoid, one-year tapered, along with immunosuppressants. Total cholesterol significantly increased in T1 vs. T0 and T2. LDL exhibited the same trend, while triglycerides increased in both T1 and T2 vs. T0. No difference was found in HDL. A significant hsCRP decrease was detected at T1 and T2 vs. T0, but not between T2 and T1. Moreover, we found a significant reduction in ESR at T2 compared with T1 and T0 and at T1 compared to T0. Hypertensive patients presented a pronounced increase in lipids, while inflammation reduced slowly compared to normotensives. Our data suggest that the increase in cholesterol and LDL in T1 is a consequence of glucocorticoids. These data can be useful in the evaluation of both CV diseases and lipid metabolism, which are closely related to vessel inflammation.

## 1. Introduction

Granulomatosis with polyangiitis (GPA) is a small–medium vessel-necrotizing vasculitis [1] classified in the spectrum of anti-neutrophil-cytoplasmic-antibody (ANCA)-associated vasculitis (AAV) [2]. The prevalence of GPA ranges from 2.3 to 146.0 cases per million persons, with an incidence of 0.4 to 11.9 cases per million persons/year [3]. Known as Wegener’s granulomatosis and lately renamed at the 2012 Chapel Hill Consensus Conference (CHCC) [4], the cause of GPA is not well understood. However, etiopathogenesis has been ascribed at least partly to ANCA [5]. Most cases (80–90%) are attributed to cytoplasmic-ANCA (c-ANCA) directed against proteinase 3 (PR3) in neutrophil granulocytes, whereas the remaining cases are attributed to perinuclear-ANCA (p-ANCA) against myeloperoxidase [1]. ANCA activates neutrophils, causing their enhanced adherence to endothelium and degranulation that can damage endothelial cells and create systemic inflammation. ANCA is 66% sensitive and 98% specific for GPA and is present in 80–90% of patients with active multisystemic disease [2,5]. Approximately 10–20% of patients with GPA are ANCA-negative, and the reason for the absence of ANCA remains unclear [1]. Patients with GPA present nonspecific symptoms for weeks or months, such as fever, malaise, myalgias, arthralgias, and weight loss, without evidence of specific organ involvement [6]. Prodromal symptoms are followed by a specific organ involvement due to necrotizing granulomatous inflammation most frequently in the upper (sinusitis, crusting rhinitis, saddle nose deformity, otitis media, mastoiditis, hearing loss) and lower (lung nodules, pulmonary interstitial capillaritis, alveolar hemorrhage) respiratory tract, in small to medium vessels (systemic necrotizing vasculitis), and in the kidney (necrotizing glomerulonephritis) [7,8]. Although the life expectancy and control of symptoms of GPA patients have improved with immunosuppressive therapies [2], atherosclerosis has now emerged as a significant morbidity [9,10], and its progression may lead to the occurrence of atherosclerotic cardiovascular disease (ASCVD). This has already been detected in other vasculitises [11] and autoimmune diseases [12,13], underlining the increased risk of ASCVD and the poor predictive value of the existing score. For this reason, early subclinical atherosclerosis results may be an important field of investigation in the treatment of GPA patients. Inflammation burden and changes in lipid profile during patients’ follow-up are involved in the progression of atherosclerosis. The purpose of this study is to evaluate lipid profile and ASCVD risk in GPA patients during follow-up.

## 2. Materials and Methods

### 2.1. Study Population

We retrospectively evaluated 63 patients relating to the Immunology clinic of the Unit of Internal Medicine, ‘Guido Baccelli’ of the Policlinico of Bari, Italy, who received a diagnosis of GPA between 1 January 2006 and 31 December 2021. Diagnoses was re-evaluated according to the recent international guidelines [1], and only patients with confirmed diagnosis were studied. We excluded patients who experienced disease worsening within two years of diagnosis. We also excluded patients that were already on statin treatment. Thus, we included 36 patients (18 females and 18 males, aged 54.73 ± 17.20) (Table 1).

Patients were evaluated at diagnosis (T0), and at first (T1) and second year (T2) of follow-up. All patients were treated with a high-dose glucocorticoid at diagnosis followed by a one-year tapering, associated with another immunosuppressant. None of the patients were affected by alcohol addiction or registered a positive Alcohol Use Disorders Identification Test (AUDIT) [14].

Patients completed a questionnaire and underwent a comprehensive physical examination, with systolic blood pressure (SBP), diastolic blood pressure (DBP), and heart rate (HR). Blood samples obtained from all patients were analyzed for the complete blood count, low/high-density lipoproteins and triglycerides. In patients with triglyceride levels over 400 mg/dl, we dosed the value of low-density lipoprotein [15]. Additional laboratory tests included erythrocyte sedimentation rate (ESR), C-reactive protein (hsCRP) level, uric acid, glycaemia, lactate dehydrogenase (LDH), alkaline phosphatase (ALP), ferritin, iron, as well as protein electrophoresis, anti-nuclear antibodies (ANA), and ANCA. Estimated glomerular filtration rate (eGFR) was calculated for each patient with CDK-EPI formula [16].

Demographic characteristics as well as clinical and laboratory findings were recorded.

We investigated the Apulian regional patient database of the healthcare system, Edotto^©^ (Exprivia, Molfetta, Italy), to identify the date of death of patients lost to follow-up.

The study protocol was part of the retrospective study on the evaluation of cardiovascular (CV) risk score in internal medicine approved by the Ethics Committee of the Policlinico University of Bari Medical School (protocol ID n.6645/20), and it conformed to the good clinical practice guidelines of the Italian Ministry of Health and the ethical guidelines of the Declaration of Helsinki, as revised and amended in 2004. Informed consent was waived due to the retrospective nature of this study.

### 2.2. Disease Activity and CV-Specific Risk Score

GPA-specific items must be evaluated to assess the diagnosis [17]. Multiple tools have been suggested to achieve this aim. First, we needed to focus on the patients’ symptoms, and the onset and progression of the disease. During physical examination, specific signs related to GPA were evaluated, such as joint swelling, skin rashes, or abnormalities in the ears, nose, or throat. Moreover, to evaluate inflammatory activity, laboratory tests were used: ESR, hsCRP, white blood cell (WBC) count, neutrophil to HDL ratio (NHR), and ANCA, specifically the PR3-c-ANCA subtype [18]. As a surrogate marker of neutrophil activity, we used the ratio between neutrophils and lymphocytes (NLR). This marker’s results were useful in the evaluation of immune cell involvement during cardiovascular disease (CVD) [11,19]. The NHR is a calculated value representing the ratio between neutrophil count and high-density lipoprotein (HDL) cholesterol levels in the blood. It can be used as a marker of systemic inflammation and CV risk [20]. Furthermore, urine tests were used to evaluate kidney involvement (the presence of red blood cells, protein, or other abnormalities).

Radiological imaging techniques were applied to assess the involvement of organs, identifying the presence of granulomas, inflammation, or structural abnormalities. Tissue biopsy served to evaluate disease diagnosis, but it is not common in follow-up of GPA [21].

Some scoring systems and disease activity indices have been developed to assess the severity and activity of GPA.

### 2.3. Birmingham Vasculitis Activity Score (BVAS)

We used the BVAS version 3 as an indicator of disease activity [22]. The BVAS provides a standardized and systematic approach to assess the extent and severity of vasculitis-related symptoms and organ involvement. The scoring system consists of various items that assess specific clinical manifestations, such as constitutional symptoms, organ-specific symptoms, and laboratory findings. Each item is evaluated and assigned a score based on its severity or presence. The scores for all individual items are then summed to calculate the total BVAS score, which represents the overall disease activity. A higher score indicates more active and severe disease [22]. For each patient, we evaluated this score at T0, T1, and T2.

### 2.4. European Society of Cardiology CV Risk Score

The European Society of Cardiology (ESC) risk score is a tool used to estimate an individual risk of developing CVD within the next 10 years [23]. The ESC CV risk score, called SCORE2, considers several key risk factors for CV disease, including age, gender, smoking status, blood pressure, total cholesterol level, diabetes, and is an important predictor of future CV events. The scoring system assigns points to each risk factor based on its impact on CV risk. The points are then summed to calculate a total risk score. The resulting score represents the estimated probability or percentage risk of experiencing a CV event, such as a heart attack or stroke, within the next 10 years. SCORE2 is useful in assessing an individual’s overall CV risk and guiding treatment decisions. It is important to note that the SCORE2 does not assess the vascular damage due to vasculitis, but it only assesses the CV risk based on the above parameters [12]. We used SCORE2 or SCORE2-OP where appropriate, according to the age of patients.

### 2.5. Statistical Analysis

This was carried out using SPSS (version 21, IBM, Armonk, NY, USA) while graphs were made using Prism (version 6.0, GraphPad Software, Boston, MA, USA). Kolmogorov–Smirnov test was performed to evaluate distribution of values. Data are presented as mean ± SD or median and interquartile range [IQR] where appropriate. Friedman test was used to evaluate the trend over time and Tukey’s multiple comparisons test was a second-step evaluation. The distribution of dichotomous values was analyzed using Chi-squared test. *p*-values are shown only for statistically significant comparisons. Survival comparison was made with the log-rank method (presented as Kaplan–Meier curves). *p* values of <0.05 were considered significant.

## 3. Results

### 3.1. Population Characteristics and Organ Damage at Diagnosis

About half (55%) of patients were hypertensive; thus, 5% of them were treated with ACE2 inhibitors (ACE2is), 27% with angiotensin receptor blockers (ARBs), 23% with calcium antagonist (CA), and 14% with beta blockers (BBs), in particular, propranolol and bisoprolol (Table 1). Ten patients were affected by diabetes and treated with metformin, only two patients with associated insulin, none with SGLT2i or GLP-1 (Table 1). At T0, all patients started therapy with glucocorticoids, 31% of them in association with azathioprine, 36% with cyclophosphamide, and 14% with methotrexate (Table 1). By analyzing the localization of GPA at T0, it becomes apparent that it was mostly localized in the ear, nose, and throat (ENT) (82%), 59% had lung involvement, and 41% eye implications (Table 1).

### 3.2. Arterial Hypertension Influences the Metabolic and Inflammatory State

We selected patients with hypertension (n. 20) and evaluated the same parameters described in Table 2 at the different time points. A significant increase in WBC at T2 vs. T1 and T0 was identified (*p* = 0.026 and *p* = 0.037, respectively), along with a reduction in L at T2 vs. T0 (*p* = 0.031) and a growth of N at T2 vs. T0 (*p* = 0.001). This resulted in a significant spread of NLR at T1 and T2 (*p* = 0.015) vs. T0 (*p* = 0.005) (Table 3). Furthermore, a significant decrease in hsCRP was found at T2 vs. T0 (*p* = 0.029) and in ESR at T2 (*p* = 0.015) and at T1 (*p* = 0.034) vs. T0 (Table 3). A different condition is shown in the group of non-hypertensive patients. WBC was not significantly higher at the different time points, but a significant reduction in N at T1 and T2 (*p* = 0.003) vs. T0 (*p* = 0.001), and T2 vs. T1 (*p* = 0.006) was observed. No differences were found in lymphocytes between the different time points. This resulted in a great reduction in NLR at T1 vs. T0 (*p* = 0.021), of hsCRP at T1 vs. T0 (*p* = 0.034) and vs. T2 (*p* = 0.038), and in ESR at T1 and at T2 vs. T0 (*p* = 0.0001 and *p* = 0.038, respectively) (Table 4).

With regard to kidney function, in hypertensives, T2 uric acid was significantly increased vs. T1 (*p* = 0.024) and T0, (*p* = 0.024) (Table 3). On the contrary, in non-hypertensive patients, a reduction in creatinine was observed at T2 vs. T1 (*p* = 0.017) and a significant increase in eGFR at T1 vs. T0 and T2 (*p* = 0.007 and *p* = 0.026, respectively) with a decrease at T2 vs. T0 (*p* = 0.049) (Table 4).

### 3.3. Metabolic and Inflammatory Parameters

**Figure 1:**
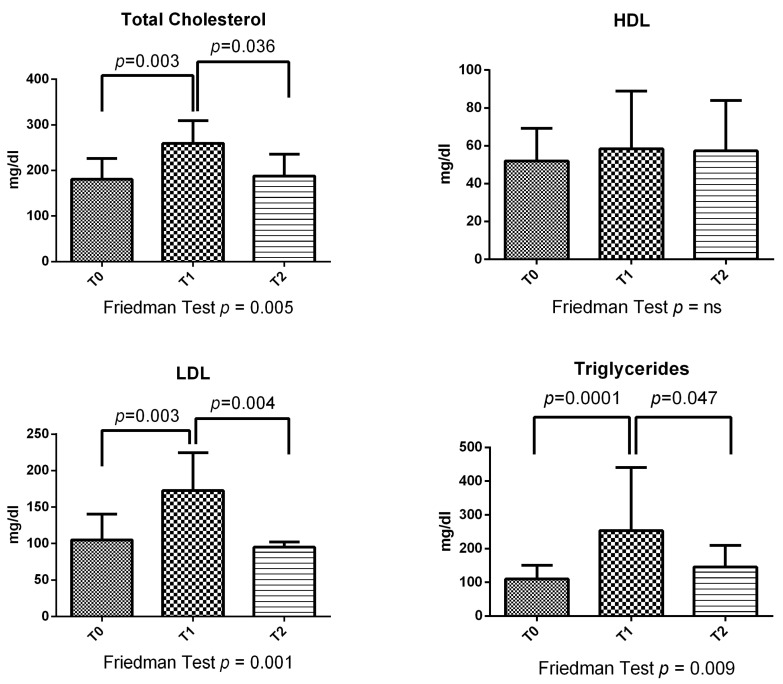
Lipids change at different time points in all 36 GPA patients.

Considering all 36 GPA patients, at T1, follow-up total cholesterol increased compared to baseline (*p* = 0.003) and T2 (*p* = 0.036) (Figure 1). LDL presented the same trend (T1 vs. T0 *p* = 0.003; T1 vs. T2 *p* = 0.004) (Figure 1). Triglycerides increased compared to baseline in T1 (*p* = 0.0001) and T2 (*p* = 0.047), while T2 and T1 overlapped (Figure 1).

**Figure 2:**
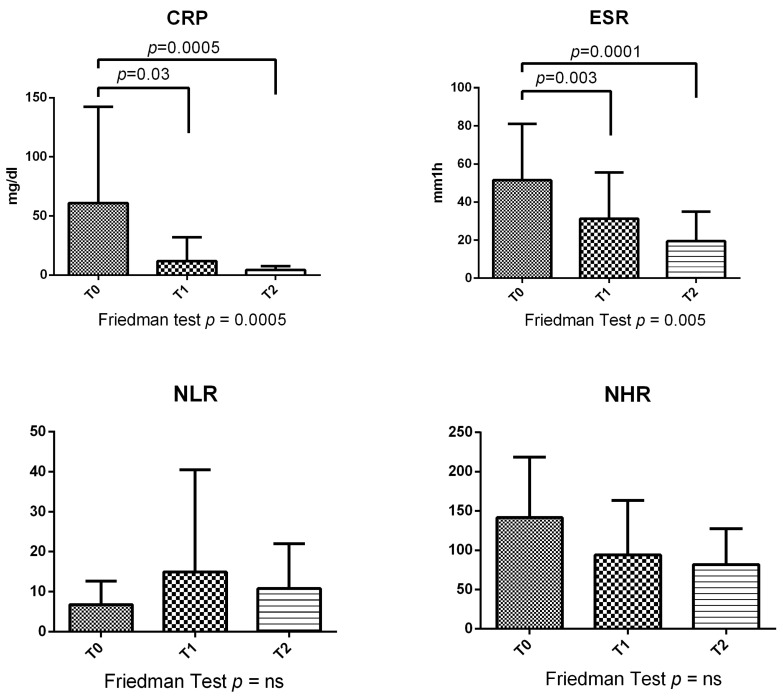
Inflammatory parameters at different time points in all 36 GPA patients, in particular hsCRP, ESR, neutrophil-to-lymphocyte ratio (NLR), and neutrophil-to-HDL ratio (NHR).

With regard to inflammatory parameters, at T1 and T2, a significant decrease in hsCRP was detected compared to T0 (*p* = 0.03 and *p* = 0.0005, respectively) (Figure 2). Similarly, ESR was significantly reduced at T2 (*p* = 0.0001) and at T1 (*p* = 0.003) compared with T0 (Figure 2). Finally, no differences in NLR at different time points in the general population were observed.

**Figure 3:**
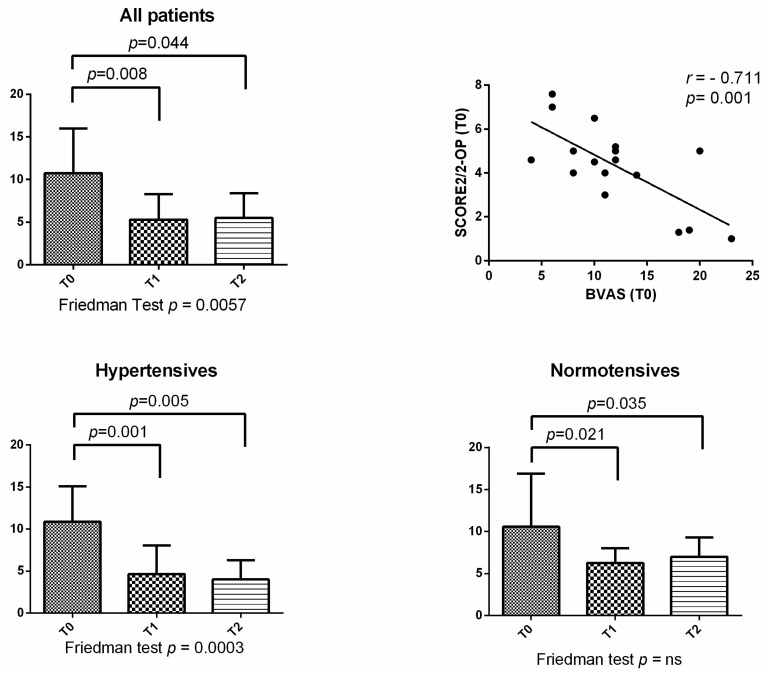
Birmingham vasculitis activity score (BVAS) score evaluated at different time points in all 36 GPA patients and its correlation to SCORE2/2-OP in T0, as well as values in hypertensives and in normotensives.

Analyzing the BVAS score during the different time points, it significantly decreased at T1 and T2 compared to T0 (*p* = 0.008 and *p* = 0.044, respectively) in all patients, and considering only hypertensives or normotensives as well (Figure 3). Moreover, we did not observe a change in BVAS in hypertensives vs. non–hypertensives (Supplementary Figure S1).

If we consider GPA patients with hypertension, a critical increase in total cholesterol was observed at T1 vs. T0 (*p* = 0.002), with a great increase in LDL at T1 vs. T0 (*p* = 0.001) and in T1 vs. T2 (*p* = 0.021) (Table 3).

A reduction in HDL at T2 vs. T0 (*p* = 0.042) was seen (Table 3). Moreover, a significant increase in triglycerides was detected at T1 vs. T0 (*p* = 0.0001) and at T1 vs. T2 (*p* = 0.010) (Table 3).

Finally, if we consider GPA patients without hypertension, a significant increase in total cholesterol was found at T1 vs. T0 (*p* = 0.005), with an increase in HDL at T1 (*p* = 0.002) and T2 (*p* = 0.017) vs. T0, and a reduction in LDL at T2 vs. T1 (*p* = 0.008) and T0 (*p* = 0.009). A increase in triglycerides was found at T1 (*p* = 0.038) and T2 (*p* = 0.0001) vs. T0 (Table 4).

### 3.4. Diagnostic Delay, Population Survival, and CV Disease

**Figure 4:**
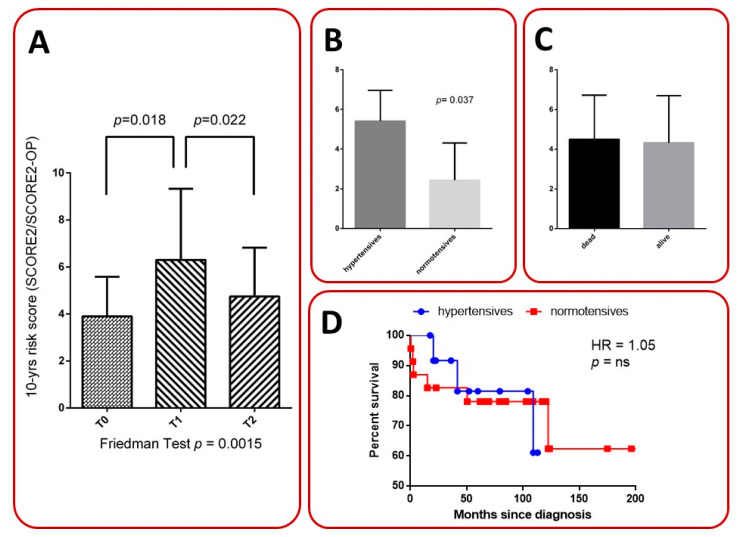
Cardiovascular (CV) risk score at 10 years at different time points in all 36 GPA patients (panel **A**), and at the time of diagnosis according to arterial hypertension history (panel **B**) or outcome (panel **C**). Panel **D** shows percentage of survival and hazard ratio in hypertensives vs. normotensives.

The majority of patients investigated in this study experienced a diagnostic delay, from 3 to 120 months. At baseline, one patient was receiving treatment for chronic atrial fibrillation. During the follow-up period, none presented a new CVD or a worsening of that previously described. We calculated the risk score in all patients affected by GPA, and it significantly increased at T1 compared to T0 and T2, as shown in Figure 4. Moreover, we calculated this score at T0 in patients according to survival outcome, and no difference was observed. Instead, we found an increased risk at 10 years, comparing hypertensives and normotensives (Figure 4). Furthermore, we analyzed the survival in GPA patients, comparing hypertensives with normotensives. The data revealed a hazard ratio greater than 1.5 that suggested a 50% increase in the risk of death in normotensives vs. hypertensives, despite no relevant differences between the two groups (*p* > 0.05) found in the survival analysis (Figure 4D).

## 4. Discussion

GPA shows defective immune-regulatory responses to environmental insults such as infections or autoantigens followed by excessive production of Th1 and Th17 cytokines (IL-17, TNF, and IFN-gamma). Pro-inflammatory cytokines led to the development of an inflammatory granulomatous vascular lesion [2].

The correlation between atherosclerosis and vasa inflammation burden has been analyzed, revealing its significant role in the increase in atherosclerosis itself, cardiovascular risk, and cardiovascular events [24].

Evidence in the general population demonstrates that LDL-C is the most important causal risk factor for ASCVD [25], leading to higher CVD risk [26]. Therefore, within the analysis of the progression of subclinical atherosclerosis of GPA patients, variations in the patients’ lipid profiles have to be taken into consideration, and lipid screening for periodic assessment of CVD risk is recommended for patients [23]. Even so, a study of 29 patients with Wegener’s granulomatosis underlined that traditional risk factors cannot explain the increase in cardiovascular risk in patients with BVAS of ≤ 1 [27]. Patients with rare autoimmune diseases show altered lipid metabolisms, which could differ based on the underlying disease [28]. However, few data and little evidence regarding lipid profile in the AAV exist, especially regarding lipid level variations during treatment. Thus, with regard to associations between lipid levels, and endothelial cell dysfunction and damage [29], understanding lipid profile variation in GPA is important.

Oral glucocorticoids are the most used for GPA patients for remission induction and maintenance [30]. Although glucocorticoids represent the cornerstone of the treatment for AAV, their optimal dosing has not yet been assessed at present, resulting in significant variability in clinical practice, especially for the induction of remission [30]. There is some controversy about the right dose that should be administered to exploit both the immunosuppressive and anti-inflammatory effects of glucocorticoids, without a high increase in collateral effects [31].

Some studies suggest that low doses of glucocorticoids might mitigate the toxicity of the treatment, while maintaining the anti-inflammatory effects, but their data are not always accepted by physicians due to the risk of a poor prognosis of an uncontrolled disease [31]. Glucocorticoids influence the immune system and its function. Furthermore, glucocorticoids have other collateral effects related to high cumulative doses: osteoporosis, CV disease, and gastrointestinal bleeding [32].

The variations in lipid profile (total cholesterol, LDL, and triglycerides) in the first year of follow-up should be related to glucocorticoid treatment needed for remission induction and maintenance of the disease. Evidence referring to changes in lipid profiles during patients’ follow-up in AAV is minimal. Wallace et al. [33] observed significant variations in lipid panels during the remission induction of AAV treatment, which was characterized by important changes in disease activity and intensive immunosuppression. The changes were mainly related to serum concentrations of total cholesterol, LDL-C, and apolipoprotein B. However, they described a shortened follow-up (six months), while we collected data up to two years from diagnosis.

A study performed on 535 patients with AAV found that, after the first year following the diagnosis, the major cause of death was CV disease [34]. Moreover, another study on 1781 patients with GPA proved a higher risk of heart failure and CV outcomes after the first year from the diagnosis compared with the general population [35]. Despite these data, the CV risk in those patients is far from being determined. A survey of 106 patients with GPA suggested that their age could be a predictor of CV events at the time of diagnosis [24].

Our data underline how lipid profile increases in the first year, especially in hypertensives, increasing the risk of CV events, but during the second year, the same data decrease without lipid-lowering drugs, according to decreases in inflammation. However, differences are evident, also considering hypertensives in inflammation. In fact, in these patients, we found a slower decrease in inflammatory markers along with a faster change in lipid profile. Therefore, these changes may interfere with CV risk evaluation.

The SCORE2 currently used is validated on the general population therefore it is non-specific for GPA. This score was higher in hypertensives, but there has been no increased risk of death found compared to normotensives when analyzing survival percentages. However, data are still controversial on CV risk change in GPA. The correlation between inflammation and change in metabolic/lipid profile is also testified by our findings despite a controversial presentation. In particular, BVAS is inversely related to CV risk in all patients. Moreover, it decreases during follow-up, resulting in a higher decrease in hypertensives. The increase in Hb values observed during the different time points could be related to the control of the inflammation. Furthermore, kidney function increases in the first year in non-hypertensives, despite it not changing in hypertensives. A possible explanation of this finding may be related to a double damage occurring in hypertensives. In these patients, better control of disease may not change the kidney damage. On the contrary, in normotensives, kidney failure is only associated with vasculitis. Thus, when GPA is treated and under control, there is an increase in filtration rate. All these data could be related to the high-dose glucocorticoid therapy that these patients take for vessel inflammation.

This study has potential limitations, such as the small sample size; on the contrary, GPA is considered a rare disease. This is also a retrospective study, including a long-time evaluation. Moreover, few patients were diagnosed via biopsy, and no histological evaluation was performed in follow-up. Finally immunosuppressive therapy may modify the lipid profile (mostly VLDL and triglycerides), making inferences about the effects of glucocorticoid therapy alone on lipid profile more difficult; however, after the first year of treatment, patients were only administered only immunosuppressants [36]. Further studies are needed to better evaluate the cardiovascular effects of vasculitis and consequent treatment.

## 5. Conclusions

Our data suggest that a change in lipid profile may not relate to inflammation. On the contrary, although the population analyzed was small and this was a significant limitation for this study, we found that the increase in total cholesterol and LDL cholesterol in the first year of follow-up should be a consequence of glucocorticoid treatment that is needed to control the spread of disease. The CV risk results increased at the time of diagnosis. Looking at metabolic risk factors, it would appear that the CV risk may worsen, especially in the first year or in hypertensive patients. However, it must be considered as a theoretical evaluation of the risk. On the contrary, real data did not show an increased incidence of CV disease, and hypertensives did not show increased mortality when GPA was detected.

These data may be helpful in evaluating CV disease and lipid metabolism due to the connection between the two parameters and vessel inflammation. However, specific patterns may be identified in order to better characterize the risk and evolution of CV in this specific group of patients.

## Figures and Tables

**Table 1:** Baseline characteristics of 36 GPA patients.

Parameter	Value
Demography	
Age (years)	54.73 ± 17.20
Females/males	18 F/18 M
Smokers	1 [2.8%]
Clinical	
SBP (mmHg)	129.4 ± 13.5
DBP (mmHg)	76.5 ± 8.1
HR (bpm)	80.7 ± 10.7
BVAS	10.7 ± 5.2
ESC SCORE2/2-OP	4.3 ± 2.2
Hypertension	20 [55%]
Diabetes	10 [27%]
Length of hospitalization (median days)	9.5 [5–16.7]
Mean survival (months)	70 [36–113]
Disease localization at T0 diagnosis	
ENT	29 [82%]
Lung	21 [59%]
Kidney	2 [9%]
Brain	7 [18%]
Abdomen	3 [18%]
Heart	3 [9%]
Skin	5 [14%]
Eyes	15 [41%]
Joints	7 [18%]
Therapy at T0 dismission	
Glucocorticoid	36 [100%]
Azathioprine	11 [31%]
Cyclophosphamide	13 [36%]
Methotrexate	5 [14%]
Angiotensin-converting enzyme 2 inhibitors	2 [5%]
Angiotensin II receptor blockers	10 [27%]
Calcium antagonist	8 [23%]
Beta-blockers	5 [14%]

BVAS: Birmingham Vasculitis Activity Score; DBP: diastolic blood pressure; ENT: ear, nose, and throat; ESC SCORE2/2-OP: European Society of Cardiology CV risk score; HR: heart rate; SBP: Systolic blood pressure.

**Table 2:** Clinical and laboratory evaluation of 36 GPA patients according to the different time points.

	T0	T1	P T1 vs. T0	T2	P T2 vs. T0	P T2 vs. T1
SBP (mmHg)	130 ± 13.5	135.7 ± 13.4	ns	133.5 ± 10.6	ns	ns
DBP (mmHg)	76.5 ± 7.9	77.1 ± 7.2	ns	78.5 ± 6.6	ns	ns
HR (bpm)	80.7 ± 10.5	84.4 ± 11.1	ns	74.4 ± 9.3	ns	0.043
Hb (mg/dL)	11.9 ± 1.4	12.8 ± 1.7	0.032	14.5 ± 1.6	0.0001	0.013
MCV (fL)	84.6 ± 7.1	84.5± 9.3	ns	85.6 ± 11.2	ns	ns
WBC (×10^3^/dL)	8.69 ± 3.83	8.18 ± 4.26	ns	9.50 ± 4.39	ns	ns
N (×10^3^/μL)	6.47 ± 0.98	5.79 ± 0.13	ns	6.00 ± 1.94	0.018	ns
L (×10^3^/μL)	1.52 ± 0.79	1.70 ± 1.05	ns	9.48 ± 2.12	ns	ns
E (×10^3^/μL)	1.04.2 ± 0.08	0.16 ± 0.11	ns	0.27 ± 0.08	0.0001	ns
M (×10^3^/μL)	0.54 ± 0.19	0.42 ± 0.21	ns	0.51 ± 0.31	ns	ns
PLT (×10^3^/μL)	231 ± 7.9	299 ± 15	ns	193 ± 100	0.025	0.048
Creatinine (mg/dL)	2.1 ± 3.1	1.2 ± 0.1	ns	0.9 ± 0.2	ns	ns
eGFR (ml/min)	78.9 ± 30.6	83.3 ± 20.8	ns	81.6 ± 17.3	ns	ns
Uric acid (mg/dL)	3.9 ± 0.5	4.7 ± 0.6	ns	7.5 ± 0.4	0.001	0.025

DBP: diastolic blood pressure; E: eosinophils; eGFR: estimated glomerular filtration rate; Hb: hemoglobin; HR: heart rate; L: lymphocytes; M: monocytes; MCV: mean corpuscular volume; N: neutrophils; PLT: platelets; SBP: systolic blood pressure; WBC: white blood cells. ns = no significance.

**Table 3:** Clinical and laboratory evaluation of 20 GPA patients with hypertension according to the different time points.

	T0	T1	P T1 vs. T0	T2	P T2 vs. T0	P T2 vs. T1
SBP (mmHg)	136.5 ± 11.7	138.3 ± 12.6	ns	139 ± 6.9	ns	ns
DBP (mmHg)	76.5 ± 8.6	78.3 ± 7.1	ns	80 ± 6.6	ns	ns
HR (bpm)	83.2 ± 11.2	88 ± 9.1	ns	78 ± 6.2	ns	0.023
Hb (mg/dL)	11.3 ± 2.1	12.9 ± 2.1	0.038	13.6 ± 1.5	ns	ns
MCV (fL)	87.5 ± 6.1	89.5 ± 4.5	ns	96.2 ± 8.2	ns	ns
WBC (×10^3^/dL)	9.09 ± 3.07	8.25 ± 3.18	ns	14.38 ±4.59	0.037	0.026
N (×10^3^/μL)	6.94 ± 1.08	5.978.3 ± 1.66.4	ns	12.28 ± 2.17	0.001	ns
L (×10^3^/μL)	1.47 ± 0.88	1.74 ± 0.14	ns	0,59 ± 0.82	0.031	ns
NLR	7.5 ± 5.8	22.6 ± 31.6	0.015	20.8 ± 16.2	0.005	ns
E (×10^3^/μL)	100 ± 100	90.7 ± 57.7	ns	0.16 ± 0.12	ns	ns
M (×10^3^/μL)	0.54 ± 0.15	0.52 ± 0.09	ns	0.16 ± 0.24	0.001	0.001
PLT (×10^3^/μL)	252 ± 89	241 ± 55		124 ± 43.2		0.021
Creatinine (mg/dL)	1.8 ± 2.5	1.1 ± 1.1	ns	1.5 ± 1.3	ns	ns
eGFR (mL/min)	68.6 ± 28.7	65.3 ± 16.5	ns	56 ± 20.4	ns	ns
Uric acid (mg/dL)	5.2 ± 1.2	5.2 ± 0.2	ns	7.5 ± 0.6	0.024	0.024
Total cholesterol (mg/dL)	177.1 ± 47.7	306 ± 53.6	0.002	120 ± 18.6	ns	ns
HDL (mg/dL)	49.4 ± 16.6	36.8 ± 15.9	ns	32.2 ± 7.1	ns	0.042
NHR (cel/μL)	156.4 ± 86.1	154.1 ± 77.5	ns	145 ± 48.3	ns	ns
LDL (mg/dL)	94 ± 41.1	221.4 ± 51.2	0.001	132.5 ± 36.8	ns	0.021
Triglycerides (mg/dL)	115.8 ± 48.5	357.2 ± 138.7	0.0001	95 ± 28.8	ns	0.010
hsCRP (mg/L)	57.1 ± 82.3	13.6 ± 23.1	ns	3.1 ± 1.4	0.029	ns
ESR (mm/h)	62.7 ± 36.2	45.7 ± 20.7	ns	26.2 ± 18.1	0.015	0.034

hsCRP: C reactive protein; DBP: diastolic blood pressure; E: eosinophils; eGFR: estimated glomerular filtration rate; ESR: erythrocyte sedimentation rate; Hb: Hemoglobin; HDL: high-density lipoprotein; HR: heart rate; L: lymphocytes; LDL: low density lipoprotein; M: monocytes; MCV: mean corpuscular volume; N: neutrophils; NHR: neutrophil-to-lymphocyte ratio; NLR: neutrophil-to-lymphocyte ratio; PLT: platelets; SBP: systolic blood pressure; WBC: white blood cells. ns = no significance.

**Table 4:** Clinical and laboratory evaluation of 16 GPA patients without hypertension (normotensives) according to the different time points.

	T0	T1	P T1 vs. T0	T2	P T2 vs. T0	P T2 vs. T1
SBP (mmHg)	119.1 ± 8.7	120 ± 6.6	ns	120 ± 7.9	ns	ns
DBP (mmHg)	76.6 ± 7.1	70.2 ± 5.1	ns	75 ± 5.7	ns	ns
HR (bpm)	77 ± 8.5	70 ± 9.1	0.016	60 ± 8.2	0.018	ns
Hb (mg/dL)	12.1 ± 1.1	12.6 ± 1.1	ns	14.7 ± 1.7	0.003	0.013
MCV (fL)	82.6 ± 7.8	78.3 ± 10.2	ns	83.1 ± 11.1	ns	ns
WBC (×10^3^/dL)	7,80 ± 4.25	8.07 ± 6.01	0.065	8.28 ± 4.04	0.055	ns
N (×10^3^/μL)	5709.6 ± 850.2	5521.9 ± 282.5	0.001	4.75 ± 0.90	0.003	0.006
L (×10^3^/μL)	1404 ± 694.2	1646.8 ± 298.7	ns	2.21 ± 1.89	ns	ns
NLR	6.8 ± 6.1	3.4 ± 0.8	0.021	7.1 ± 10.1	ns	ns
E (×10^3^/μL)	0.10 ± 0.07	0.26 ± 0.09	0.001	0.23 ± 0.07	0.0001	0.001
M (×10^3^/μL)	0.49 ± 0.20	0.27 ± 0.27	0.045	0.63 ± 0.01	ns	0.041
PLT (×10^3^/μL)	268 ± 71.7	357 ± 207	ns	210.7 ±105.6	0.007	ns
Creatinine (mg/dL)	1.1 ± 0.8	1.2 ± 0.2	0.038	0.9 ± 0.2	ns	0.017
eGFR (ml/min)	86.4 ± 35.6	109.1 ± 52.8	0.007	81.6 ± 21.4	0.049	0.026
Uric acid (mg/dL)	4.6 ± 1.6	4.3 ± 0.2	ns	3.7 ± 5.3	ns	ns
Total cholesterol (mg/dL)	182.4 ± 45.5	213 ± 58.6	0.005	208.3 ± 28.9	ns	ns
HDL (mg/dL)	55.4 ± 17.6	80.1 ± 15.9	0.002	72.5 ± 14.2	0.017	ns
NHR (cel/μL)	120.8 ± 60.2	73.8 ± 50.2	ns	81.7 ± 45.9	ns	ns
LDL (mg/dL)	115.6 ± 26.1	124.1 ± 31.2	ns	95 ± 8.1	0.008	0.009
Triglycerides (mg/dL)	105.1 ± 35.9	146 ± 18.7	0.038	231 ± 15.2	0.0001	ns
hsCRP (mg/L)	309.1 ± 436.8	5.5 ± 9.6	0.034	5.3 ± 3.9	0.038	ns
ESR (mm/h)	56 ± 26.3	14 ± 9.7	0.0001	17 ± 9.3	0.0001	ns

hsCRP: C reactive protein; DBP: diastolic blood pressure; E: eosinophils; eGFR: estimated glomerular filtration rate; ESR: erythrocyte sedimentation rate; Hb: hemoglobin; HDL: high-density lipoprotein; HR: heart rate; L: lymphocytes; LDL: low-density lipoprotein; M: monocytes; MCV: mean corpuscular volume; N: neutrophils; NHR: neutrophil-to-lymphocyte ratio; NLR: neutrophil-to-lymphocyte ratio; PLT: platelets; SBP: systolic blood pressure; WBC: white blood cells. ns = no significance.

## Data Availability

The raw data supporting the conclusions of this article will be made available by the authors, without undue reservation.

## Supplementary Materials

The following supporting information can be downloaded at: https://www.mdpi.com/article/10.3390/metabo13101053/s1, Figure S1: BVAS changes in different time points in hypertensives (blue line) and normotensives (red line). No significant difference was found at each timepoint.
