# Supplementary material for: Changes in Lipids in Granulomatosis with Polyangiitis Relates to Glucocorticoids and History of Hypertension
Source: Metabolites. 2023 Oct 6;13(10):1053. doi: 10.3390/metabo13101053 (PMC10608943; doi:10.3390/metabo13101053)
Supplement: Supplementary file 1 [file metabolites-13-01053-s001.zip › metabolites-2599326-supplementary.pdf]

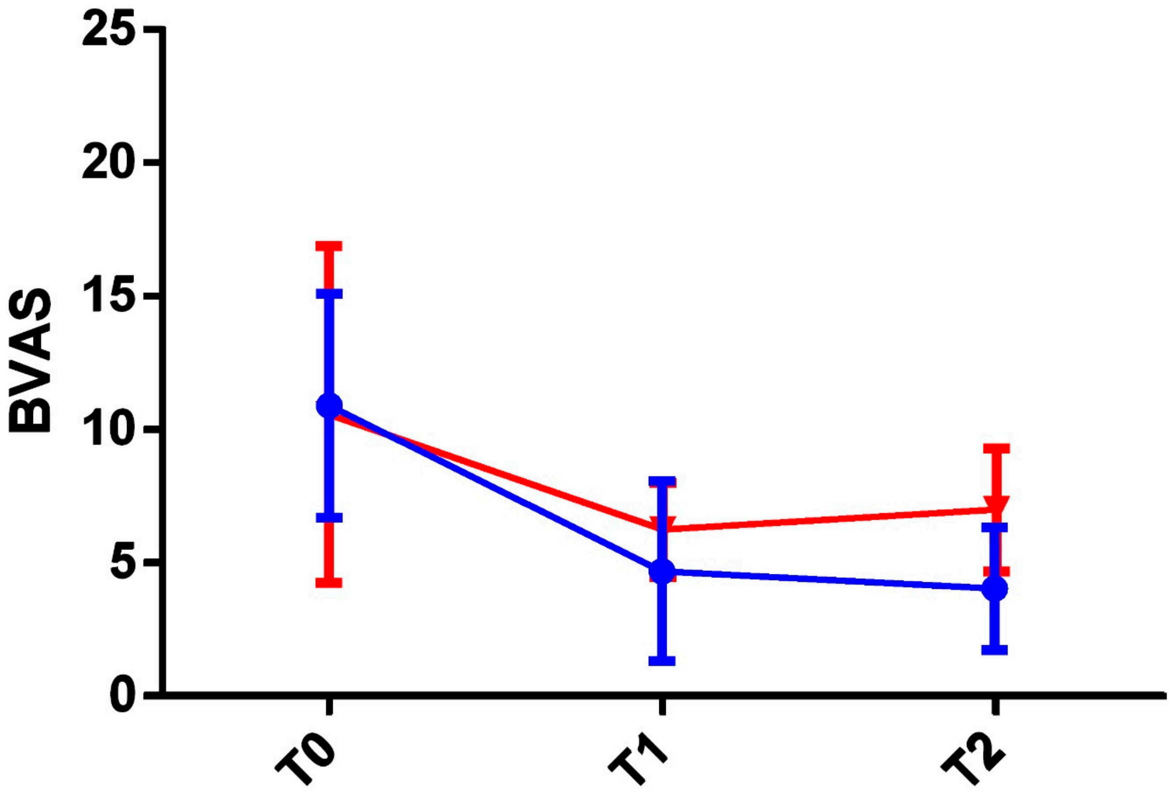

Supplementary Figure S1. BVAS changes in different time points in hypertensives (blue line) and normotensives (red line). No significant difference was found at each timepoint.
